
JOURNAL INFORMATION
==============================
NLM Title Abbreviation: ScientificWorldJournal
Journal ID: ScientificWorldJournal
NLM Journal ID: 101131163
Journal ID: TSWJ

The Scientific World Journal

ISSN: 2356-6140
EISSN: 1537-744X
Publisher: Wiley

ARTICLE INFORMATION
==============================
PMCID: PMC6009669
PMID: 29973827
DOI: 10.1100/tsw.2002.52
Article ID: 636385
Article version: 1
Subjects: Short Report

Encoding Selectivity of a Transmembrane Channel

Stroud Robert M. *
Nollert Peter
Miercke Larry J.W.
Harries William E.C.
O'Connell Joe
S-964 UCSF Medical School, USA
*Robert M. Stroud: stroud@msg.ucsf.edu
Publication date: 2002
Electronic publication date: 2002 Apr 13
Volume: 2
First page: 111
Last page: 111
Copyright: Copyright © 2002 Robert M. Stroud et al.
Copyright year: 2002
License: This is an open access article distributed under the Creative Commons Attribution License, which permits unrestricted use, distribution, and reproduction in any medium, provided the original work is properly cited.
License URL: https://creativecommons.org/licenses/by/3.0/

==============================
Advances in Gene Technology: The Genome and Beyond –
Structural Biology for Medicine (Proceedings of the 2002 Miami
*Nature Biotechnology* Winter Symposium)
*TheScientificWorld* 2002, 2(S2), 111
ISSN 1532-2246; DOI 10.1100/tsw.2002.52

### ENCODING SELECTIVITY OF A TRANSMEMBRANE CHANNEL

Robert M. Stroud*, Peter Nollert, Larry J.W. Miercke, William E.C. Harries, Joe O'Connell
S-964 UCSF Medical School
stroud@msg.ucsf.edu

Membrane channel proteins of the aquaporin family are integral plasma membrane proteins that are highly selective for permeation of specific small molecules, with absolute exclusion of ions including protons or OH- anions and charged solutes, and without dissipating the electrochemical potential across the cell membrane. There are 11 human family members. The family includes channels that conduct water, called aquaporins, and channels that conduct glycerol or other linear polyalcohols (aquaglyceroporins), and ion conducting channels. The *E. coli* glycerol facilitator (GlpF) was cloned, expressed, purified, and crystallized with its primary permeant substrate glycerol. The crystal structure of GlpF, determined by multiple isomorphous replacement at 2.2 Å resolution illustrate the basis for its selectivity. Glycerol molecules G1, G2, G3 line up in an amphipathic channel in single file. In the narrow selectivity filter of the channel, the glycerol alkyl backbone is wedged against a hydrophobic corner and successive OH groups form hydrogen bonds with a pair of acceptor, and donor atoms. These hydrogen bonds serve to orient the CHX-OH groups within the narrower regions of the channel, and explain the stereo, and enantio selectivity measured for this channel. Two conserved -Asn Pro Ala- motifs meet in the center of the membrane and form a conserved interface between two gene-duplicated segments that each encode three and one half membrane spanning helices around the channel. This structure elucidates the mechanism of selective permeability for linear carbohydrates and suggests how ions and water are excluded. A functional assay was developed, and shows that the channel is both stereo- and enantio-selective. The conservation of sequence shows how the water channels of the same family conduct water and exclude glycerol.

The nature of the channel directly shows how it rejects conduction of protons. This is measured in assays, and tested in collaboration with the Shulten group using molecular mechanics. A comparison of features of the channel itself, and of the space through the center of the tetrameric arrangement of proteins suggests that it is the ion channel in AQP6. Mutations made in the sequence to mimic aquaporins AQP1, and AQP6 lead us to distinguish the basis for water vs. glycerol selectivity, and provide us with a high resolution, tunable structure of an ion channel.

This functional, and structural analysis provides arguably the most detailed bases for understanding of the selelctivity of channels for permeant ions, molecules, and organic molecules.

### REFERENCES

1. Fu, D., Libson, A., Miercke, L., Weitzman, C., Nollert, P., Krucinski, J., and Stroud, R. (2000) Science 290, 481–486.
2. Nollert, P., Harries, W.E.C., Fu, D., Miercke, L.J.W., and Stroud, R.M. (2001) *FEBS Lett.* 504(3), 112–117.
